# A Sound of Relief or a Sound of Panic: A Case Report on Female Urethral Sounding

**DOI:** 10.7759/cureus.78316

**Published:** 2025-01-31

**Authors:** Grace Kuroki, Zachariah Taylor, Max Ahn

**Affiliations:** 1 Urology, Drexel University College of Medicine, Philadelphia, USA; 2 Urology, Main Line Health, Lankenau Medical Center, Wynnewood, USA

**Keywords:** dangerous sexual practices, female urethral dilatation, foreign body insertion, foreign body removal, sounding

## Abstract

Medical urethral sounding is a common practice used to dilate the urethra. However, recreational practice comes with additional risks and perceived social stigma. This article describes a case of a middle-aged female patient inserting a 12-cm sounding device into her urethra during sexual intercourse with her partner that became lodged in her bladder. After bedside cystoscopy failed, she needed to be taken to the operating room for surgical removal. We discuss the medical complications of insertion, in both the male and female urethras, in addition to commentary on the threat that delay in treatment poses to the individuals. Safe recreational sounding practices are mentioned to best prepare the patient and physician for these situations.

## Introduction

Examining the realm of “extreme” sexual practices invites conversation about the current understanding of the ways people seek pleasure. From asphyxiation to urethral sounding, there are several sexual practices that could result in serious bodily harm. Urethral sounding is the practice of insertion of an object or liquid into the urethra [1]. While sounding is often used in urological procedures for dilatation of urethral strictures for placement of urethral catheters or to access the bladder endoscopically, it is used recreationally for sexual pleasure [1]. The practice of sounding has a risk of foreign objects becoming stuck in the urethra or potentially migrating into the urinary bladder, thus increasing the risk of infection, injury, or trauma [2]. An injury or retained foreign object from urethral sounding may be characterized by hematuria, urinary tract infections (UTIs), or pain [3]. However, patients may delay treatment due to social stigma or fear of judgment [3].

Sparse data is available detailing the prevalence of urethral sounding in the population, with most of the reported literature being from case reports [2]. Furthermore, most of this data reports on male sounding, with only a few reports on female sounding [4]. We present a rarely reported case of a female with a retained foreign object in the bladder after urethral sounding.

## Case presentation

A 45-year-old female patient presented to the emergency department with a retained foreign body about four hours after urethral sounding with her partner during sexual intercourse. She regularly practiced urethral sounding; however, this was the first time she needed medical attention. In the emergency department, she reported an inability to fully empty her bladder but denied any pain or discomfort. She also had no signs of UTI.

**Figure 1:**
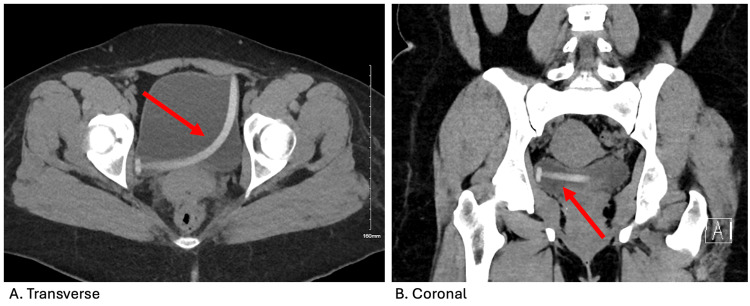
(A and B) CT scans (transverse and coronal views) of the foreign body located in the bladder, indicated with a red arrow.

**Figure 2:**
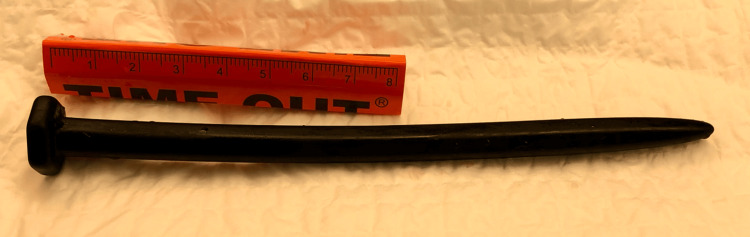
Foreign body after surgical retrieval from the bladder.

The patient underwent a CT scan, which showed a foreign object localized in her bladder (Figures 1A, 1B). A Foley catheter was inserted due to increasing suprapubic pain and fullness at the bedside, and about 700 cc of urine was collected. Initially, bedside flexible cystoscopy attempted to snare the object using a wire basket but was ultimately unsuccessful. The patient was then recommended rigid cystoscopy in the operating room under monitored anesthesia care. The patient was positioned in lithotomy and prepped using a standard surgical technique. A 21 French cystoscope was advanced through the urethral meatus, and a pan cystoscopy was performed. The approximately 12-cm silicon foreign body was found in the bladder, and graspers were used to remove the object through the urethra (Figure 2). No bladder or urethral perforations or damage was noted. Following the procedure, the patient was discharged home that same day.

## Discussion

Urethral sounding devices have been dated back over 2,000 years; they have been found among the Greek and Roman ruins [5]. The advancement of these devices aligns with the early ages of metal discoveries such as copper, bronze, lead, tin, and iron [5]. The devices were used to discover a urethral stricture or the presence of a foreign body. The name “sound” was coined due to the thin metal rod making a particular “sound” when hitting a stone in the bladder [5]. Presently, urethral sounding is still used as a means for stricture dilatation or accessing the bladder. However, sounding has also expanded to a recreational practice for sexual stimulation or pleasure, with kits readily available for purchase across the internet [6]. Little data is available on how this practice began, and even today, few papers are published about this topic, due to presumed social stigma and lack of reporting [7]. Even fewer papers are reported on females who participate in recreational practice [8].

Physicians and patients should be aware of the potential risks associated with urethral sounding and prepare for the complications, should a problem arise. Acutely, the risk of bleeding, urethral rupture, bladder perforation, and UTI increases due to the entry of bacteria into the urethra [1,7]. Men may have an increased risk of urethral rupture due to the length of their urethra, while women may experience higher rates of bladder perforation [3,7]. Chronic or delayed complications include recurrent UTI, urethral stricture, or the creation of false passages [7]. In addition, the perceived social stigma associated with urethral sounding may pose more risks to the patient. Oftentimes, a patient will delay treatment for a sounding complication, which increases the risk of calcifications, bacterial infections, and mucosal tears [2,7,9].

Most importantly, physicians should be educating their patients on safe sounding practices. Patients should be advised to avoid recreational urethral sounding if they have a history of UTI, are immunocompromised, are pregnant, or have known structural conditions in their urethra [1,10]. Patients should be encouraged to use sterile objects while practicing, to avoid the increased risk of bacterial infection leading to UTI, or to thoroughly clean and properly store their sounds in between use [2,10]. It is not recommended to share sounds between individuals or sexual partners, to decrease the risk of sharing any viruses or bacteria. Patients should be encouraged to speak openly with their providers about their usage and to seek medical attention should any part of their devices dislodge the body. It is important for physicians to properly assess the well-being of the person participating in urethral sounding, to ensure that they are acting within their normal psychological behavior and are not exhibiting any signs of mental illness [1,8].

## Conclusions

Physicians need to be equipped with knowledge about urethral sounding and its prevalence in both men and women, despite the lack of reported cases. Physicians need to address the topic professionally and delicately, as there is a large range of possible emotions surrounding the topic of sexual pleasure, including “extreme” practices such as urethral sounding. With honest patient histories and a nonjudgmental environment, the physician and patient can discover the best care plan for the individual and assess their personal needs.
